# The Mitochondria-Associated ER Membranes Are Novel Subcellular Locations Enriched for Inflammatory-Responsive MicroRNAs

**DOI:** 10.1007/s12035-020-01937-y

**Published:** 2020-05-25

**Authors:** Wang-Xia Wang, Paresh Prajapati, Peter T. Nelson, Joe E. Springer

**Affiliations:** 1 Sanders-Brown Center on Aging, University of Kentucky, 800 S. Limestone, Lexington, KY 40536 USA; 2 Spinal Cord and Brain Injury Research Center, University of Kentucky, Lexington, KY 40536 USA; 3 Pathology & Laboratory Medicine, University of Kentucky, Lexington, KY 40536 USA; 4 Neuroscience, University of Kentucky, Lexington, KY 40536 USA

**Keywords:** Mitochondria-associated ER membrane, Subcellular, Neurodegeneration, Traumatic brain injury, microRNA, miR-146a

## Abstract

The mitochondria-associated endoplasmic reticulum (ER) membranes (MAMs) are specific ER domains that contact the mitochondria and function to facilitate communication between ER and mitochondria. Disruption of contact between the mitochondria and ER is associated with a variety of pathophysiological conditions including neurodegenerative diseases. Considering the many cellular functions of MAMs, we hypothesized that MAMs play an important role in regulating microRNA (miRNA) activity linked to its unique location between mitochondria and ER. Here we present new findings from human and rat brains indicating that the MAMs are subcellular sites enriched for specific miRNAs. We employed subcellular fractionation and TaqMan® RT-qPCR miRNA analysis to quantify miRNA levels in subcellular fractions isolated from male rat brains and six human brain samples. We found that MAMs contain a substantial number of miRNAs and the profile differs significantly from that of cytosolic, mitochondria, or ER. Interestingly, MAMs are particularly enriched in inflammatory-responsive miRNAs, including miR-146a, miR-142-3p, and miR-142-5p in both human and rat brains; miR-223 MAM enrichment was observed only in human brain samples. Further, mitochondrial uncoupling or traumatic brain injury in male rats resulted in the alteration of inflammatory miRNA enrichment in the isolated subcellular fractions. These observations demonstrate that miRNAs are distributed differentially in organelles and may re-distribute between organelles and the cytosol in response to cellular stress and metabolic demands.

**Electronic supplementary material:**

The online version of this article (10.1007/s12035-020-01937-y) contains supplementary material, which is available to authorized users.

## Introduction

Many physiological functions are regulated via the mitochondria-associated ER membranes (MAMs). The MAMs are specific ER domains that tether the mitochondria to regions of the ER and play a role in lipid synthesis, calcium transport and homeostasis, mitochondria dynamics, and regulating autophagosome and inflammasome formation [1–12]. It is thus not surprising that disturbances in MAM-regulated biological processes affect a wide range of normal cellular processes. For example, dysfunction of the MAMs has been implicated in an array of human diseases, including neurodegenerative diseases such as Alzheimer’s disease (AD), Parkinson’s disease (PD), and amyotrophic lateral sclerosis (ALS) [13–21]. However, there are many unanswered questions as to how the mitochondria and ER physiological processes are regulated in relation to MAMs, and what additional functions may be attributed to this cell domain. In the current study, we propose a previously undescribed feature of MAMs in the brain, related to the presence of microRNAs (miRNAs).

MiRNAs are small non-coding RNAs that regulate gene expression post-transcriptionally through mRNA degradation or translational inhibition. Many miRNAs are evolutionarily conserved, and collectively, they are thought to regulate as much as two-thirds of all human genes [22, 23]. MiRNA dysregulation has been implicated in many human diseases including neurodegenerative diseases and neurotrauma [24–26].

Much has been learned about miRNA processing, but many questions also remain. Following transcription in the nucleus, a miRNA precursor transcript is exported to the cytoplasm and then further processed to produce a mature ~ 22 nucleotide miRNA. Execution of miRNA function is carried out in association with an Argonaute (AGO) protein-containing complex, and the gene regulatory activities are widely believed to occur mostly in the cytoplasm. There is now accumulating evidence documenting the presence of miRNA/AGO complexes in various organelles, including the nucleus, ER, and mitochondria [27–29]. This implies the existence of currently unknown mechanisms for miRNA subcellular distribution, trafficking, and inter-organelle interactions. However, our understanding of miRNA intracellular trafficking is very limited, particularly in the mammalian brain. Therefore, studies aimed at revealing miRNA interactions with organelles under normal and stressed conditions will be critical for advancing our understanding of miRNA biology in the central nervous system.

We reported previously that several inflammatory-responsive miRNAs (e.g., miR-146a, miR-142-3p, and miR-142-5p) are preferentially associated with mitochondria isolated from rat hippocampus, and that these miRNAs display a mitochondria-to-cytoplasm compartmental shift following a severe TBI [30, 31]. To gain better insight into inter-organelle miRNA distribution and function, we performed subcellular fractionation of brain tissue from rapidly autopsied human frontal cortices and rat cortices, and then analyzed miRNA and AGO protein expression in the highly enriched mitochondria, MAMs, ER, and cytosol subcellular fractions. We also examined the subcellular distribution pattern of these miRNAs in cortical samples of rats treated with a mitochondrial uncoupling agent and also in rats subjected to a severe TBI, which is associated with a rapid compromise in mitochondrial function. Our findings indicate that the MAMs are particularly enriched for inflammatory miRNAs. Furthermore, the re-distribution of inflammatory miRNAs following cellular insults may suggest a potential mitochondria–MAM–ER axis for miRNA trafficking.

## Materials and Methods

**Fig. 1:**
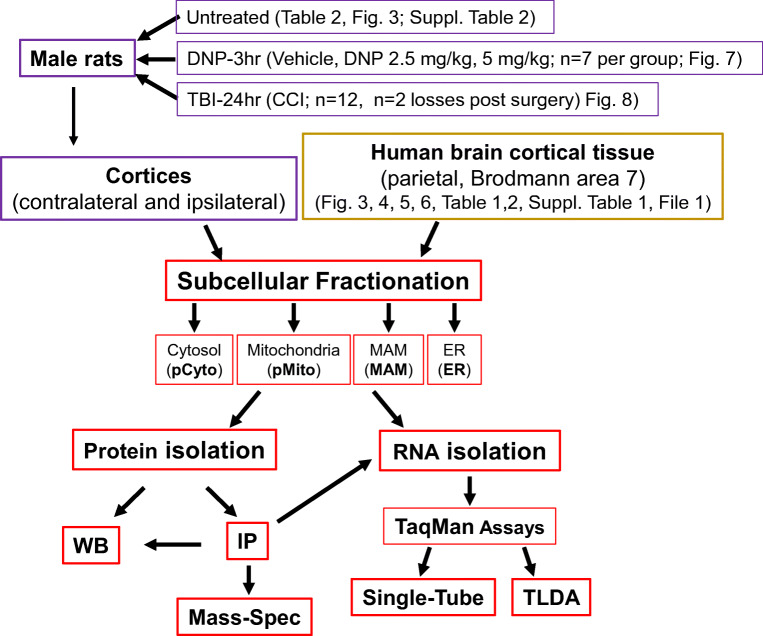
Overall experimental procedures conducted in the study. The chart outlines the major experimental procedures and the animal numbers/losses for the animal experiments. The associated figures/tables are indicated

The overall experimental procedures conducted in this study are summarized in Fig. 1.

### Human Brain Tissue

All procedures and protocols related to procurement of the human brain specimens were approved by the University of Kentucky Institutional Review Board (IRB no. 44009). Human brain tissues were obtained from the University of Kentucky Alzheimer’s Disease Center (UK-ADC) biobank. The recruitment and the informed consent process has been detailed previously [32, 33]. Potential volunteers who indicated a willingness to participate in the UK-ADC research program received an introductory personal letter explaining the critical need for understanding the cause of AD, followed by a visit with a center staff to provide information about the research in the UK-ADC. Each potential volunteer was then contacted by telephone and provided an opportunity to ask questions about study procedures and autopsy. With mutual agreement, a home visit was made with the individual to review and sign the informed consent document. All human neocortical (parietal, Brodmann area 7) specimens were obtained at autopsy with postmortem intervals (PMI) less than 4 h (except case no. 2, Table 1). The specimens were immediately immersed in ice-cold IB-B buffer (225 mM mannitol, 25 mM sucrose, and 30 mM Tris–HCl, pH 7.4) containing protease inhibitors (Halt™ Protease and Phosphatase Inhibitor Cocktail (100×), 78440, Promega) and kept on ice.

**Table 1:** Demographic and neuropathologic information on human research subjects

Case no.	Age at death	Sex	Last clinical dx	MMSE	Brain wt (g)	PMI (h)	Braak	AD	Other
1	79	F	Demented	20	1230	1.9	2	No	Late
2	90	M	Normal	25	1340	9.5	1	No	
3	94	M	Normal	25	1110	1.5	2	No	
4	91	F	Normal	29	1100	2.7	2	No	
5	94	M	MCI	24	1250	2.5	5	Yes	
6	97	M	MCI	29	1110	1.8	2	No	CVD

### Animal Studies: Controlled Cortical Impact Injury and Mitochondria Uncoupling

All animal procedures used in this study conformed to the US *Public Health Service Policy on Humane Care and Use of Laboratory Animals* and the National Institutes of Health *Guide for the Care and Use of Laboratory Animals* and were approved by the University of Kentucky’s Institutional Animal Care and Use Committee (IACUC protocol no.: 2014-1300). At the present time, controlled cortical impact (CCI) and mitochondria uncoupling animal models established in our labs have been carried out in only male rats; therefore, only male animals were used in this study. Young adult male Sprague–Dawley rats (RRID:MGI:5651135) weighing 250–300 g (~ 2 months old, Harlan Laboratories, IN) at the time of surgery were housed two per cage in Allentown PC10198HT cage (259 mm × 476 mm × 209 mm; 910 cm^2^; top MBT1019HT; wire bar lid WBL1019RSMD) for 1 week prior to experimentation and maintained in a temperature-controlled vivarium room with free access to food and water. Animals were arbitrarily assigned to treatments of TBI or sham surgery. The procedures for the surgical and CCI injury used in our lab have been described previously [31, 34]. Briefly, animals were anesthetized with 4% isoflurane and placed in a stereotaxic frame (David Kopf, Tujunga, CA) prior to surgery. Anesthesia was maintained with 2.5% isoflurane delivered via a nose cone throughout the surgical procedure. Using sterile procedures, the skin was retracted, and a craniotomy (6 mm) was made lateral to the sagittal suture centered between the bregma and lambda. After careful removal of the skull cap to preserve integrity of the dura, the exposed brain was injured using a pneumatically controlled impacting Precision Systems and Instrumentation Head Impactor device (Fairfax Station, VA). A 5-mm-diameter rod tip was used to compress the cortex at a velocity of 3.5 m/s to a depth of 2.0 mm, which results in a severe injury to the cortex [35–37]. Following surgery, a piece of Surgicel (Ethicon, Inc.) was laid over the dura, the skull-cap replaced, and a thin coat of dental acrylic spread over the craniotomy site to stabilize the skull-cap and allowed to dry before closing the overlying skin with surgical staples. The core body temperature of the animals was maintained at 37 °C throughout the surgical procedures and recovery period. Post-operative analgesics were not given since the experimental animals typically show no untoward side effects once they have recovered from anesthesia, and resume normal eating, drinking, and grooming patterns. In addition, pre- or post-injury administration of analgesics can influence the nature of brain injury, as well as mitochondrial function and microRNA activity [38–40]. Furthermore, at 1–3 h after brain injury, rats are assessed for signs of pain and any rats receiving a level 2 score on the assessment scale (provided by veterinarians of Division of Laboratory Animal Resources) were euthanized immediately. CCI experiments were done between 9 and 12 am.

Animals were euthanatized at 24 h following CCI and the ipsilateral (Ipsi) and contralateral (Contra) cortices rapidly removed for isolation of the subcellular fractions. The decision for selecting 24 h post-CCI in this study was guided by the well-described observation that a loss of mitochondria function and inflammatory responses are significantly elevated at this time point [41–43]. A total of 12 animals were initially subjected to TBI and 2 animals were lost during the post-surgical period leaving a final total of 10 animals. For the mitochondria uncoupling experiments, animals (*n* = 7 per group, no animals were excluded or loss during the experiments) received an intraperitoneal injection of either 2.5 mg/kg or 5 mg/kg 2,4-dinitrophenol (DNP) or dimethyl sulfoxide (DMSO) vehicle. Animals were monitored for adverse effects following the injection, and no pain was observed in these animals. Animals were euthanatized at 3 h following treatment and the cortices rapidly dissected for subcellular fractionation. The treatment time and uncoupling DNP dosage were selected following the study reported by the Sullivan laboratory, which showed that uncoupling effectively takes place in rat brain mitochondria at this time point and is well-tolerated [44]. DNP experiments were done between 9 and 11 am.

At the end of the experiments, the animals were euthanized by exposure to carbon dioxide using a carbon dioxide chamber until the absence of movement and respiration followed by immediate decapitation.

### Subcellular Fractionation Procedure

**Fig. 2:**
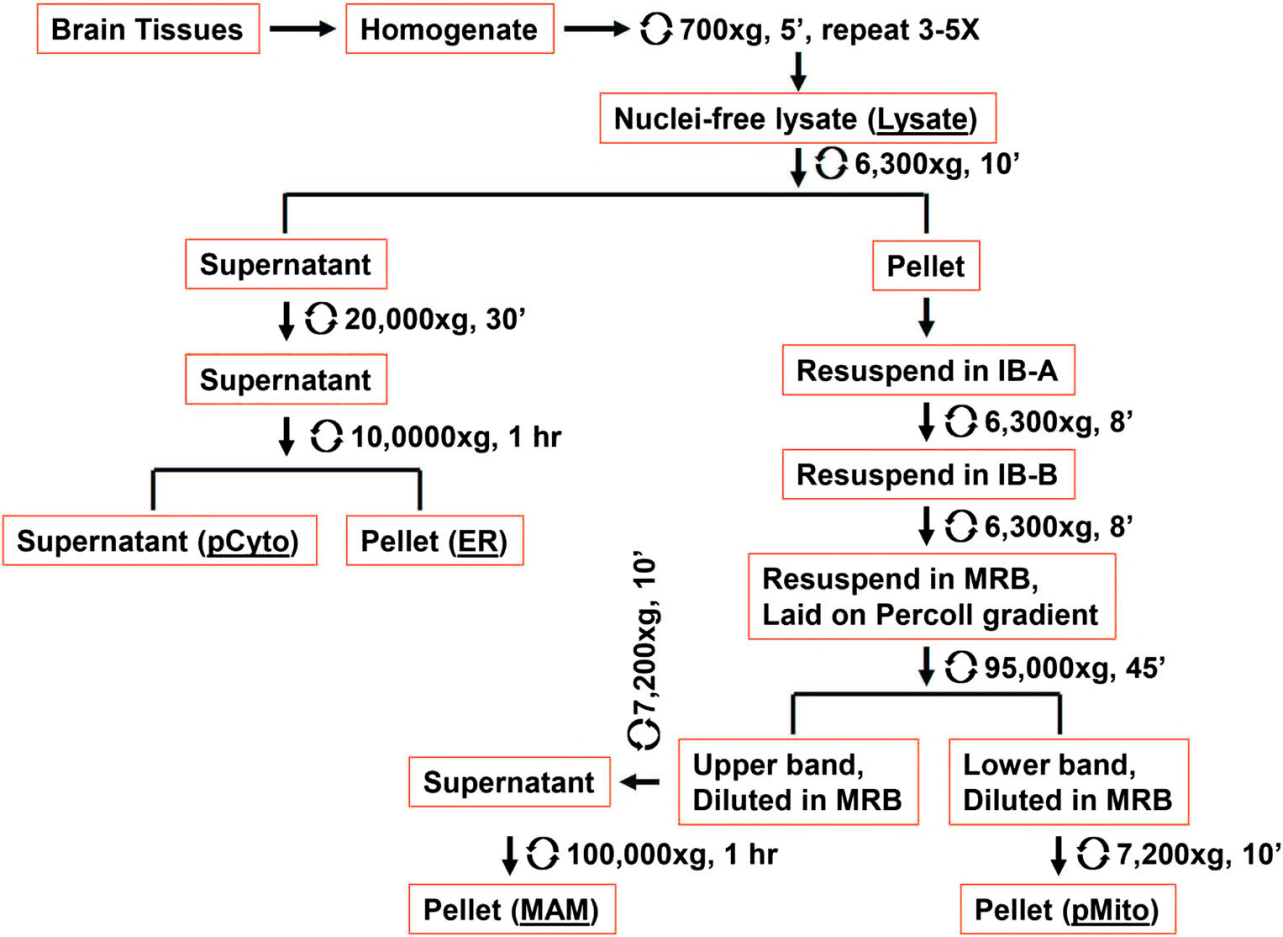
Schematic flowchart of brain tissue subcellular fractionation procedure. Subcellular fractionation is carried out using series of centrifugations. The speed and time needed for each subcellular fractions are indicated in the flowchart

The procedures used for subcellular fractionation of freshly autopsied human brain tissue or rat brain tissue were a modification of two well-established published protocols [45, 46] to accommodate for brain tissue (Fig. 2). Briefly, immediately following removal of a 5–10-g portion of human cortex or one-half of rat cortical hemisphere, the tissue was immersed in an ice-cold IB-B buffer (225 mM mannitol, 25 mM sucrose, and 30 mM Tris–HCl, pH 7.4) containing protease inhibitors. Meninges and visible blood vessels were removed. For the human brain tissue samples, gray and white matter were separated from each other, with only the gray matter being used in this study. Brain tissue was cut into small pieces and transferred to a pre-cooled glass/Teflon Potter homogenizer. HB buffer (225 mM mannitol, 25 mM sucrose, 0.5% BSA, 0.5 mM EGTA, and 30 mM Tris–HCl, pH 7.4) plus protease inhibitors was added at a volume of approximately 3 ml buffer per 0.5-g tissue weight and homogenization performed on ice. The homogenate was then transferred to a 5-ml tube and centrifuged at 700×*g* for 5 min at 4 °C. The supernatant was collected in a fresh tube, and the pellet again was gently homogenized using additional buffer and centrifuged as above. The supernatant from the second centrifugation was combined with the initial supernatant fraction and this process repeated 2–3 times until the buffer to tissue weight ratio reached 10:1 (i.e., 5 ml supernatant was obtained from 0.5-g tissue). The combined supernatant fractions were further centrifuged at 700×*g* for 5 min at 4 °C to remove residual nuclei/tissue debris, and the process was repeated until no significant pellet was observed and the resulting supernatant was collected as post-nuclear lysate (*Lysate*). A crude mitochondria/MAM fraction was obtained by centrifugation of the post-nuclear lysate at 6300×*g* for 10 min at 4 °C. The supernatant was removed and saved for ER and cytosol isolation. The crude mitochondrial/MAM pellet was gently transferred to a 7-ml loose fitting glass homogenizer using a 1-ml pipette tip with an enlarged opening to minimize any shearing of the pellet. The pellet was then gently resuspended in IB-A buffer (225 mM mannitol, 25 mM sucrose, 0.5% BSA, and 30 mM Tris–HCl, pH 7.4) using 3–5 gentle strokes with a glass homogenizer, and the homogenate was centrifuged at 6300×*g* for 8 min at 4 °C. The above process was repeated using IB-B buffer, and the pellet was resuspended in 2 ml MRB buffer (250 mM mannitol, 0.5 mM EGTA, and 5 mM HEPES, pH 7.4). The crude mitochondrial/MAM suspension was then carefully laid over a 30% Percoll medium solution (30% Percoll, 225 mM mannitol, 1 mM EGTA, and 25 mM HEPES, pH 7.4) and centrifuged at 95,000×*g* for 45 min at 4 °C. Two bands formed after centrifugation with the upper band being enriched with the MAM fraction, and the lower band containing a purified fraction of mitochondria. The fractions from the respective bands were collected into separate centrifuge tubes and diluted 10 times in MRB buffer followed by centrifugation at 7200×*g* for 10 min at 4 °C. Supernatant from the MAM-enriched tube was transferred to an ultracentrifuge tube and centrifuged at 100,000×*g* for 1 h at 4 °C. After centrifugation, the MAMs were then collected in a 1.5-ml Eppendorf tube and topped with MRB buffer and centrifuged at top speed in a bench-top centrifuge for 10 min at 4 °C; the pellet is MAM fraction (*MAM*). Supernatant from the purified mitochondrial fraction was discarded, and the pellet (purified mitochondria, *pMito*) was resuspended in 200-μl MRB buffer and used for further analysis. The supernatant from the crude mitochondria/MAM centrifugation step was further centrifuged at 20,000×*g* for 30 min at 4 °C. The supernatant was then subjected to ultracentrifugation at 100,000×*g* for 1 h at 4 °C with the resulting pellet containing purified ER (*ER*) and the supernatant saved as purified cytosol (*pCyto*).

### MAM, ER, Mitochondria, and Post-nuclear Total Lysate Protein Extractions

For Western blot analyses, subcellular fractions were incubated with RIPA buffer (10 mM Tris–Cl (pH 8.0), 1 mM EDTA, 0.5 mM EGTA, 1% Triton X-100, 0.1% sodium deoxycholate, 0.1% SDS, R0278, Sigma) in the presence of protease inhibitors (Halt™ Protease and Phosphatase Inhibitor Cocktail (100×), Promega) for 20 min on ice, followed by centrifugation at 10,000×*g* for 10 min at 4 °C. Supernatant was saved and the protein concentrations determined using the BCA protein assay method (Bio-Rad). For the immunoprecipitation (IP) experiments a milder IP lysis buffer (25 mM Tris–HCl, pH 8.0, 150 mM NaCl, 1% Triton X-100) was used.

### Immunoprecipitation

The procedure of co-immunoprecipitation (IP) of Argonaute (AGO) using 2A8 antibody [47] or normal mouse serum (NMS) (Rockland) has been described previously [48, 49] and was performed with some modifications. Briefly, each of the subcellular fractions was lysed in IP lysis buffer containing protease inhibitors and 0.25 unit/μl of RNasin (N2111, Promega). Supernatants were saved following centrifugation at 10,000×*g* for 10 min at 4 °C. The protein concentrations from each fraction were highly varied. For example, the protein concentration of the isolated MAM fraction from each 0.5-g tissue typically ranged from 1 to 1.7 mg/ml when MAM fractions were lysed in 200 μl buffer, which is equivalent to approximately 200 to 340 μg total protein. In contrast, the protein concentration of purified cytosol ranges from 2.5 to 3.8 mg/ml with ~ 4.5 ml total volume, which would result in approximately 11,250 to 17,100 μg total protein. For the IP experiments, each supernatant (800, 800, 200, 200, and 200 μl for Lysate, pCyto, pMito, MAMs, and ER, respectively) was divided equally between two fresh tubes labeled with either anti-AGO antibody or NMS, so that anti-AGO and NMS IP would receive equal quantity of proteins. Additional buffer was added to pMito, MAM, and ER solution to make up to 400 μl in each tube. Note that the IP experiments were conducted in order to determine the specificity of the pulldown products of anti-AGO antibody in comparison with that of NMS and not to compare AGO levels across fractions. The fractions were first incubated with either anti-AGO (2A8) antibody [47] or NMS for 1–2 h at 4 °C under gentle rotation. After the initial incubation step, a 20-μl slurry of Protein G Agarose beads (15920010, Thermo Fisher) was rinsed in PBS and equilibrated in IP lysis buffer prior to addition to each tube, and the incubation continued for additional 5–6 h. The beads were spun down and washed in 1.0 ml IP buffer for 4 times at room temperature. The beads were then transferred with additional 1.0 ml IP buffer to a fresh tube and pelleted. For protein analysis, beads were then incubated with 40 μl Laemmli sample buffer at 95 °C for 6 min to extract bound proteins. For RNA isolation, the beads were resuspended in 275 μl of IP buffer with addition of 750 μl of TRIzol™ LS. The procedure for RNA isolation is described below under the “RNA isolation from subcellular fractions” heading.

### Western Blot Analysis

The Western blotting procedure has been published previously [31]. Briefly, protein samples (10–20 μg) from each fraction were separated using 4–15% Mini-PROTEAN® TGXTM Gel (4561086, Bio-Rad). After transblotting onto nitrocellulose membranes, the blots were incubated with 5% nonfat milk for 1 h at room temperature before overnight incubation at 4 °C with the respective primary antibodies. Blots were then washed and incubated in affinity-purified peroxidase-conjugated secondary antibodies (Cat no. 115-036-062, RRID:AB_2307346, Cat no. 111-035-144, RRID:AB_2307391, Jackson ImmunoResearch, West Grove, PA; antibody dilution: 1:20,000), and the immunoreactive signals were visualized using a Chemiluminescent substrate kit (SuperSignal™ West Pico PLUS Chemiluminescent Substrate, 34,577, Pierce). The antibodies used in this study included NDUFA9 (NADH dehydrogenase [ubiquinone] 1 alpha subcomplex subunit 9, mitochondria marker, Thermo Fisher Scientific Cat no. 459100, RRID:AB_2532223; antibody dilution: 1:5000), Grp 75 (glucose-regulated protein 75, mitochondria/MAM marker, Santa Cruz Biotechnology Cat no. sc-133,137, RRID:AB_2120468; antibody dilution: 1:500), PDZD8 (PDZ domain-containing protein 8, MAM/ER marker, a kind gift from Joseph Sodroski [50]; antibody dilution: 1: 500), Calnexin (ER/MAM marker, Abcam Cat no. ab22595, RRID:AB_2069006; antibody dilution: 1:5000), α-Tubuling (cytosol marker, Santa Cruz Biotechnology Cat no. sc-8035, RRID:AB_628408; antibody dilution: 1:10,000).

### Mass Spectrometry

Mass spectrometry analysis was performed at the University of Kentucky Proteomics Core Facility. Protein products co-immunoprecipitated with either AGO or NMS were resolved by 4–12% SDS-PAGE (NP0322BOX, Thermo Fisher), stained with Sypro Ruby (50564, Lonza), and visualized using the FluoChem™ R System (ProteinSimple). Three gel pieces from each lane (MAM-IP-AGO and MAM-IP-NMS) were excised at the similar position in their respective lanes. The gel pieces were treated with dithiothreitol (DTT) reduction and iodoacetamide (IAA) alkylation and digested in-gel with trypsin before being subjecting to LC–MS/MS analysis. The LC–MS/MS data sets of the gel pieces from the same IP sample (MAM-IP-AGO or MAM-IP-NMS) were combined. Each of the combined data sets was searched with MASCOT for protein identification against a custom database containing a total of 20,349 *Homo sapiens* (Human) proteins from Uniprot (current version, downloaded 06/01/2018, http://www.uniprot.org/uniprot/?query=human&fil=organism%3A%22Homo+sapiens+%28Human%29+%5B9606%5D%22+AND+reviewed%3Ayes&sort=score).

### RNA Isolation from Subcellular Fractions

TRIzol™ LS reagent (15596018, Thermo Fisher) was used to isolate total RNA from purified mitochondria, MAMs, ER, cytosol, post-nuclear total lysate, and IP products following a modified procedure [31, 51]. RNA concentrations were determined using NanoDrop1000 Spectrophotometer (NanoDrop Technologies, Inc.).

### TaqMan® Low-Density Array Analysis of miRNA Expression in Subcellular Fractions

A customized TaqMan® Low-Density Array (TLDA) panel containing selected miRNAs associated with neurodegenerative diseases was used to analyze miRNA expression in each subcellular fractions of human samples following the protocol previously described [52]. Due to the low quantity of total RNA in the mitochondria and MAM fractions, a preamplification step was carried out for all the TLDA analysis to maintain technical consistency. Briefly, total RNA (30 ng) from each subcellular fraction was subjected to reverse transcription reaction using TaqMan® MicroRNA Reverse Transcription Kit (Thermo Fisher) with custom-pooled RT primers. An aliquot of RT product was pre-amplified using pooled preamplification primers and TaqMan® PreAmp Master Mix (Thermo Fisher). The pre-amplified products served as templates and were combined with TaqMan® Universal PCR Master Mix (No AmpErase UNG, Thermo Fisher) for the TLDA analysis. Real-time PCR and data collection was performed using ViiA™ 7 Real-Time PCR System (Thermo Fisher) using the manufacturer’s standard program (hold 2 min at 50 °C, followed by 10 min at 95 °C, then 40 cycles of 15 s at 95 °C and 1 min at 60 °C).

### TaqMan® Single-Tube miRNA and Gene Expression Assays

Single-tube TaqMan® miRNA assays (4427975, Thermo Fisher) were performed with preamplification step as previously described [52]. RT and preamp primers of tested miRNAs were first pooled to a final concentration of 0.25× and 0.2×, respectively. Equal amount (30 ng) of total RNA from each subcellular fractions was RT and preamp following manufacturer’s instructions. The RNA concentrations of samples isolated from immunoprecipitation was usually very low and the Nanodrop measurement is unable to reflect the true RNA concentration. For these IP samples, equal volume (3 μl) of RNA was used. TaqMan® gene expression assays were employed to determine the levels of glyceraldehyde 3-phosphate dehydrogenase (*Gapdh)* (*Rn01775763*) (Thermo fisher) in each subcellular fractions with equal quantities of total RNAs (100–300 ng) used. Quantitative PCR was performed with a QuantStudio™ 7 Real-Time PCR System (Thermo Fisher) using the manufacturer’s standard program (hold 2 min at 50 °C, followed by 10 min at 95 °C, then 40 cycles of 15 s at 95 °C and 1 min at 60 °C).

A list of all TaqMan® assay IDs used in this study can be found in Suppl. File 2. TaqMan® miRNA and gene expression assays either in single-tube or array format were designed to follow MIQE (Minimum Information for Publication of Quantitative Real-Time PCR Experiments) guidelines [53] according to the company’s statement.

### Data Analysis

Raw PCR Ct values were generated using QuantStudio™ Real-Time PCR Software with automatic baseline and threshold (Thermo Fisher). The data set was uploaded to Thermo Fisher Data Cloud and further analyzed using Thermo Fisher Cloud Software-Relative Quantification. PCR Ct values ≥ 35 were considered as undetectable and excluded from further analysis. An appropriate internal normalizer could not be identified due the composition of the different subcellular sources of RNA. Therefore, the Global Mean Normalization method [54] was used to normalize the TLDA or single-tube data. The relative miRNA levels or *Gapdh* mRNA quantity (RQ) in each fraction were expressed as 2^−∆Ct^ value (2 to the power of negative deltaCt).

### Experimental Design and Statistical Analysis

Power analysis considerations for animal studies: the number of animals per group was based upon power function analysis of previous data obtained in our labs and from other laboratories using identical procedures (DNP and TBI). Using this information and a confidence interval (CI) of 95% (i.e., significance level of *p* < 0.05), sample sizes (*n* = 6) were determined that would provide acceptable levels of statistical power. Estimates are based on suggestions from Keppel (Design and Analysis, Prentice Hall, NY, 1973). In all instances, the studies are designed to maximize the amount of information derived from a minimal number of animals.

In all real-time quantitative PCR experiments, we considered that a minimum of 1.5-fold change, or a Ct difference of 0.58 (fold 1.5 = 2^0.58) is needed to discern a biological meaningful difference in 2-sample *t* test. The power analysis and sample size (*n* = 3–6) for RT-qPCR experiments in this study was estimated using following excel formula: *n* = ROUNDUP((*Z* × *σ*/*E*)^2.0). The parameters were CI = 0.95, *Z* (*Z*-score) = 1.96, *σ* (standard deviation, Ct) = 0.6 (human), *σ* = 0.3 (rat), *E* (margin of error, Ct) = 0.58. No comparison (e.g., race, sex, age, or pathology) was made across the human samples related to the subcellular miRNA distribution patterns.

The Shapiro–Wilk test was completed to ensure normality for conducting parametric analysis. One-way ANOVAs and Student’s *t* tests (2-tailed) were applied (GraphPad Prism 7) to evaluate mean differences when appropriate. When the data set deviated from Gaussian distribution, the non-parametric Mann–Whitney test was employed to test for significant differences. No test was conducted for outliers and no data were excluded. The statistical method for each experiment, degree of freedom (df), and effect size (*d*) is specified in the “Results” section. Confidence interval of 95% was used in all data analysis with a *p* < 0.05 being considered statistically significant.

Blinding procedures for rat experiments: for the TBI, the surgery and brain injury procedure were performed by an individual who was not involved in any other subsequent procedures. All animals/samples were assigned with only a tail mark/number. Persons who performed brain tissue harvesting, subcellular fractionation, RNA/protein isolation, and RT-qPCR were given only sample/tube numbers. For the DNP experiments, the injection, brain tissue harvesting, and subcellular fractionation procedures were performed by the same individuals and all animals/samples assigned only numbers. RNA isolation and RT-qPCR were performed by individuals who did not have the knowledge of sample identities. Data analysis was performed by an individual who was not involved in the RT-qPCR experiments. In both TBI and DNP experiments, the sample IDs were only revealed during the data analysis. The study was exploratory; no exclusion criteria were pre-determined. No randomization was performed, and the study was not pre-registered.

## Results

### Validation of Subcellular Fractionation Procedure

**Fig. 3:**
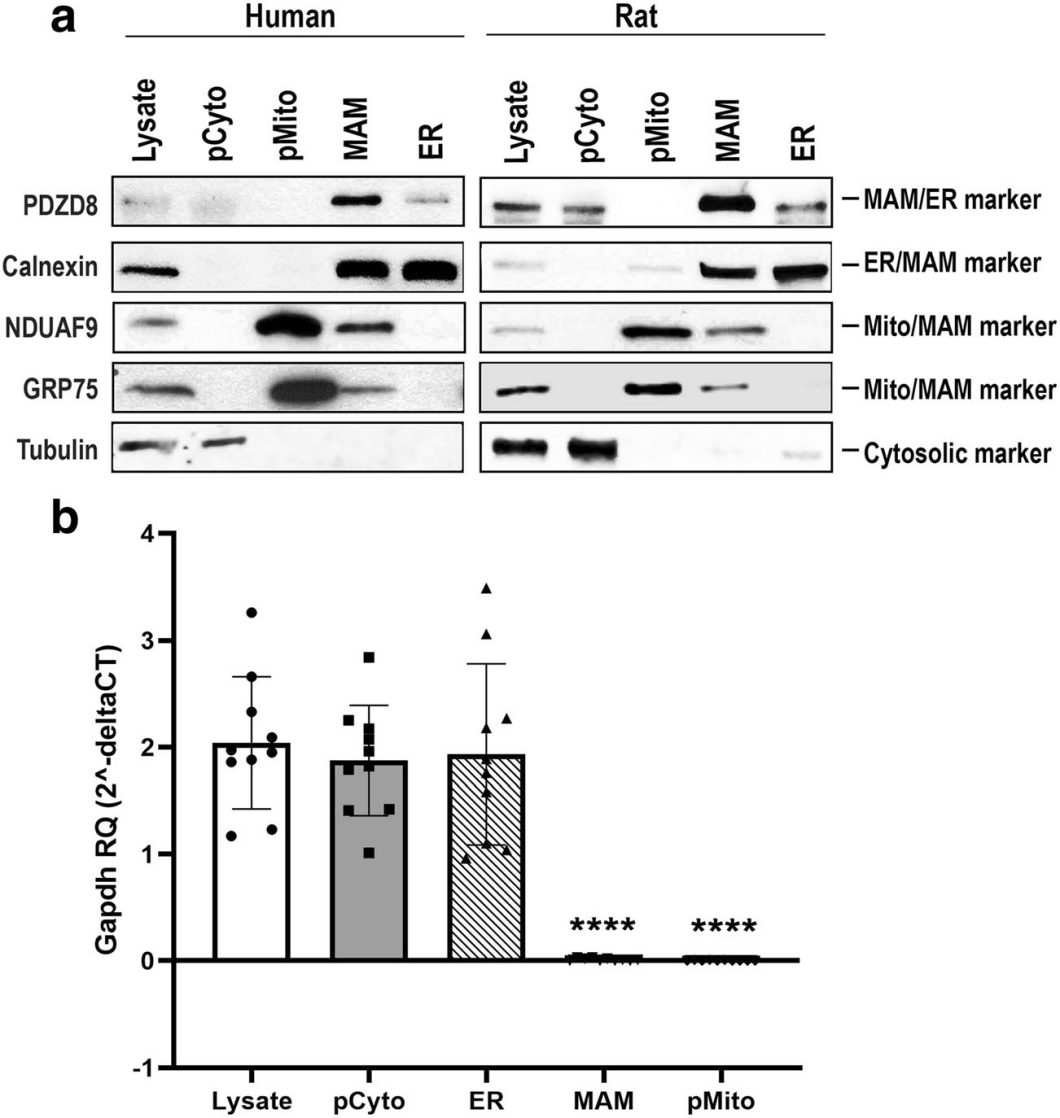
Analysis of purity/enrichment of subcellular fractions. **a** Western blot analysis of protein markers for each subcellular fraction was used to document the enrichment of the subcellular fractions isolated from human and rat cortex. **b** RT-qPCR analysis of *Gapdh* mRNA. *Gapdh* mRNA in various subcellular fractions from rat brain tissues (*n* = 10 different independent biological samples) was examined using single-tube TaqMan® assays. The raw Ct values were normalized using the global mean method and the relative quantity (RQ) expressed as RQ = 2^−∆Ct^ (RQ equals 2 to the power of negative delta Ct). A one-way ANOVA was used to analyze differences among each subcellular fraction and, when warranted, differences between individual fractions evaluated using an unpaired Student’s *t* test or Mann–Whitney test. A *p* value < 0.05 was considered being statistically significant. ****p < 0.0001, Mann–Whitney test). *Lysate* (RQ = 2.04): post-nuclear total lysate; *pCyto* (RQ = 1.87): cytosol fraction; *pMito* (RQ = 0.003): mitochondria fraction; *MAM* (RQ = 0.012): mitochondria-associated ER membrane fraction; *ER* (RQ = 1.93): ER fraction

Subcellular fractionation was conducted on freshly autopsied (PMI < 4 h) human cerebral cortices and rat brains using a modified protocol (Fig. 2, [55]). The isolated fractions were routinely examined for purity/enrichment using Western blotting with conventional organelle-specific marker proteins (Fig. 3a). The antibodies used included anti-NDUFA9 (mitochondria/MAM), anti-Grp75 (mitochondria/MAM), anti-Calnexin (ER/MAM), anti-PDZD8 (MAM/ER), and anti-tubulin (cytosol). The Western blots showed that organelle-specific protein markers were associated with their anticipated specific subcellular fractions (Fig. 3a). No known protein marker can be used to specially analyze the MAM fraction due to the unique subcellular location between the ER and mitochondria. However, the banding patterns of mitochondrial markers NDUFA9 and Grp75, ER marker Calnexin, and a MAM-associated marker PDZD8 [56] indicate that the fraction was highly enriched for MAMs. No major organellar protein bands were found in the cytosol, indicating that it was devoid of the organelles examined. In addition, we used anti-tubulin antibodies to cross-examine potential presence of cytosol in organelle fractions. Tubulin was largely absent in organelle fractions when using anti-α-tubulin antibody sc-8035 (Santa Cruz). Overall, the staining pattern of the Western blots confirmed that the fractionation protocol enabled effective isolation of highly enriched mitochondria, MAMs, ER, and cytosol, and each individual fraction was relatively free of other subcellular fractions.

### Detection of miRNAs in MAMs

As a first step to determine whether miRNAs exist in MAMs, single-tube TaqMan® assays were performed on RNA extracted from the subcellular preparations using a method developed for isolation of total RNA from mitochondrial fractions [31]. To rule out the possibility of cytosolic RNA contaminating MAMs and mitochondria fractions, the presence of *Gapdh* transcripts was examined using highly sensitive RT-qPCR analysis. *Gapdh* mRNA RT-qPCR signals were found to be more than 100-fold lower in the MAMs and more than 600-fold lower in the mitochondria fractions relative to cytosol or ER (Fig. 3b). Notably, although *Gapdh* is a cytosolic protein-encoded gene, several studies have documented the presence of *Gapdh* mRNA in association with the ER [57–61]. Our finding that *Gapdh* mRNA is observed in the ER fraction was consistent with these previous studies.

MiRNA TaqMan® assays with preamplification were employed to detect whether miRNAs were present in MAMs. The analysis revealed that several miRNAs were detected in the MAM fractions isolated from both human (Suppl. Table 1) and rat (Suppl. Table 2) cortex. These include three miRNAs, miR-146a, miR-142-3p, and miR-142-5p, that were previously identified to be highly enriched in rat hippocampal mitochondria [30, 31], as well as several other miRNAs including miR-223 and two neuronal-enriched miRNAs, miR-124 and miR-107 [62].

**Table 2:** MAM-associated miRNAs are bound in AGO complexes

	**Human cortical tissue**							
	**Case 3**				**Case 5**			
	**MAM**		**pCyto**		**MAM**		**pCyto**	
IPs	AGO	NMS	AGO	NMS	AGO	NMS	AGO	NMS
miR-107	151.8	1.0	1183.8	1.0	3.0	1.0	25.6	1.0
miR-142-3p	16.5	1.0	766.0	1.0	7.7	1.0	62.4	1.0
miR-142-5p	14.3	1.0	370.4	1.0	10.3	1.0	4.2	1.0
miR-146a	155.9	1.0	158.7	1.0	36.9	1.0	67.6	1.0
miR-223	163.1	1.0	890.7	1.0	29.0	1.0	68.4	1.0
	**Rat cortical tissue**							
	**Rat 1**				**Rat 2**			
	**MAM**		**pCyto**		**MAM**		**pCyto**	
IPs	AGO	NMS	AGO	NMS	AGO	NMS	AGO	NMS
miR-107	214.7	1.00	549.1	1.00	3.2	1.0	30.8	1.0
miR-142-3p	77.3	1.00	223.2	1.00	9.8	1.0	64.3	1.0
miR-142-5p	32.6	1.00	8.2	1.00	8.7	1.0	34.5	1.0
miR-146a	631.2	1.00	328.3	1.00	56.2	1.0	43.1	1.0
miR-223	90.1	1.00	662.6	1.00	36.0	1.0	56.8	1.0

Single-tube TaqMan® miRNA analysis of AGO and NMS IP products from MAM and cytosol fractions**.** The relative miRNA levels in AGO-IP were expressed as fold change relative to NMS-IP in the same fraction (mitochondria-associated ER membrane fraction (*MAM*) or cytosol (*pCyto*)). The fold change was calculated using the following equation: fold change = 2 − (Ct-AGO-IP − Ct-NMS-IP) (fold change equals 2 to the power of negative (Ct-AGO-IP minus Ct-NMS-IP)

### Distinct Subcellular Distribution of miRNAs in Human Cortical Tissues

**Fig. 4:**
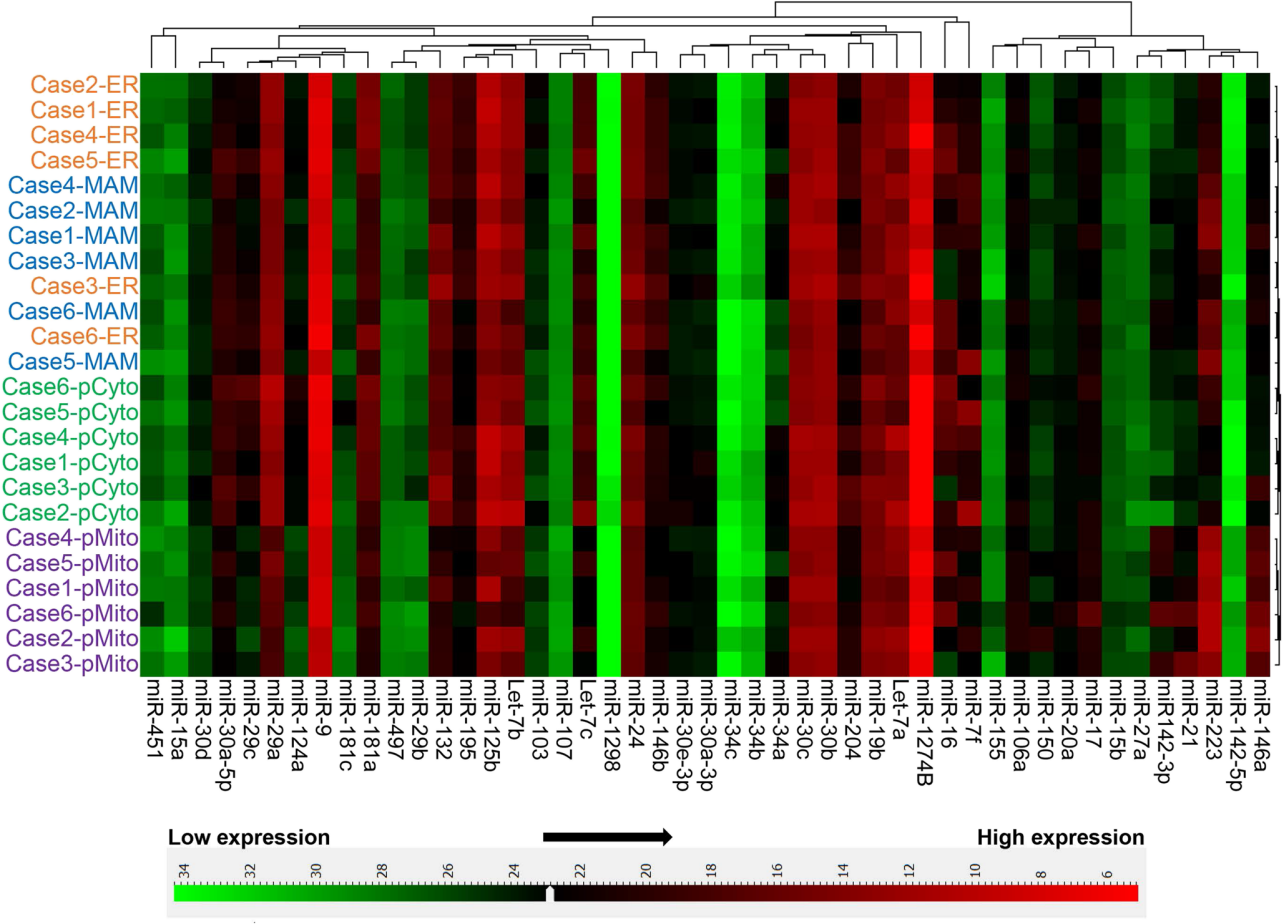
Hierarchical clustering of miRNA expression in subcellular fractions from human cortices. Cytosol (*pCyto*), mitochondria (*pMito*), *MAM*, and *ER* subcellular fractions (*n* = 6 individual human cases) were analyzed for miRNA expression using a neurodegenerative disease-focused, custom miRNA TaqMan® Low-Density Array (TLDA) panel. The global mean normalization method was used to normalize TLDA Ct values. Pearson’s distance-average linkage was applied for hierarchical clustering analysis of Ct values

Based on the findings of the single-tube TaqMan® assays, we further analyzed the presence of other miRNAs using a neurodegenerative disease-focused, custom miRNA TaqMan® Low-Density Array (TLDA) panel, which simultaneously quantified 47 miRNAs and a control small RNA U6 as described previously [52]. The TLDA analysis of the subcellular fractions of 6 human brain samples revealed that these 47 miRNAs were differentially present in the 4 subcellular fractions consisting of cytosol (*pCyto*), mitochondria (*pMito*), *MAM*, and *ER* (Fig. 4). Hierarchical clustering (Pearson’s distance-Average Linkage) showed that miRNA expression was highly clustered according to each subcellular fraction.

**Fig. 5:**
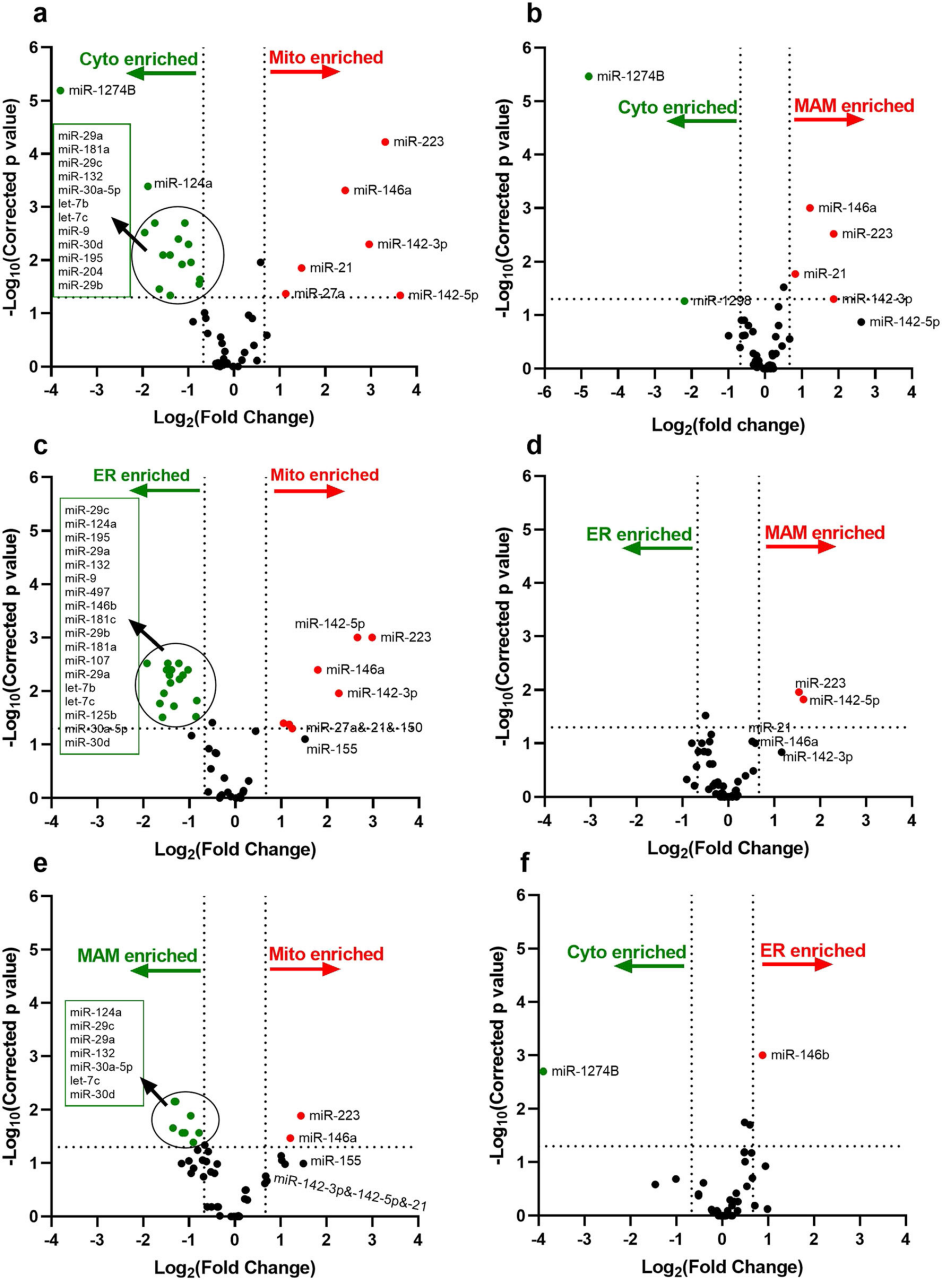
Volcano plots of miRNA expression levels in subcellular fractions isolated from human cortices. Cytosol (*pCyto*), mitochondria (*pMito*), *MAM*, and *ER* subcellular fractions (*n* = 6 individual human cases) were analyzed for miRNA expression using a neurodegenerative disease-focused, custom miRNA TaqMan® Low-Density Array (TLDA) panel. The global mean normalization method was used to normalize TLDA Ct values. Each plot provides a visual representation of the degree of miRNA enrichment in paired subcellular fractions (e.g., *Cyto* enrichment vs. *Mito* enrichment). Levels of miRNA expression in the subcellular fractions were evaluated using unpaired Student’s *t* test and Benjamin–Hochberg multiple testing to correct *p* values. A corrected *p* value < 0.05 was considered being statistically significant. The *X*-axis is the log_2_ of the fold change between the two subcellular fractions, and the *Y*-axis is the negative log_10_ of the corrected *p* value. The horizontal dash line (*Y* = 1.3) corresponds to a threshold *p* value of 0.05 (*Y* = − Log_10_(0.05) = 1.3) and the vertical dash lines corresponds to a threshold of 1.5-fold increased or decreased level (*X* = Log_2_(± 1.5) = ±0.67). In each panel, miRNAs with a significant (corrected *p* < 0.05) increase of more than 1.5-fold are colored in red and those with a significant (corrected *p* < 0.05) decrease of more than 1.5-fold are colored in green. The plots represent the comparison between: mitochondria and cytosol (**a**), MAM and cytosol (**b**), mitochondria and ER (**c**), MAM and ER (**d**), mitochondria and MAM (**e**), ER and cytosol (**f**)

We then analyzed the TLDA data by comparing the subcellular fractions in volcano plots and found that distinct fraction-specific miRNA distribution patterns were evident (unpaired Student’s *t* test and Benjamin–Hochberg multiple testing to control FDR (5%)). The previously reported rat hippocampal mitochondria-enriched miRNAs (miR-146a, miR-142-3p, and miR-142-5p) were also enriched in human brain mitochondria fractions relative to cytosol, ER and MAMs (Fig. 5a, c, e, Suppl. Table 1). In addition, miR-223, which was not enriched in rat cortical or hippocampal mitochondria [31] or MAMs (Suppl. Table 2), exhibited appreciable enrichment in human samples—both in human brain mitochondria and MAMs (Fig. 5a–d, Suppl. Table 1). Other miRNAs relatively enriched in human cortical tissue mitochondria included miR-21 and miR-27a. Intriguingly, the mitochondria-enriched miRNAs, miR-146a, miR-223, miR-21, miR-142-3p, and miR-142-5p, were also present at relatively high levels in human cortical MAM fractions compared with cytosol and ER (Fig. 5b, d, Suppl. Table 1). Finally, no significant difference between the ER and cytosol fractions was observed for these mitochondria/MAM-enriched miRNAs (Fig. 5f).

### Argonaute Protein Is Localized in the MAMs

**Fig. 6:**
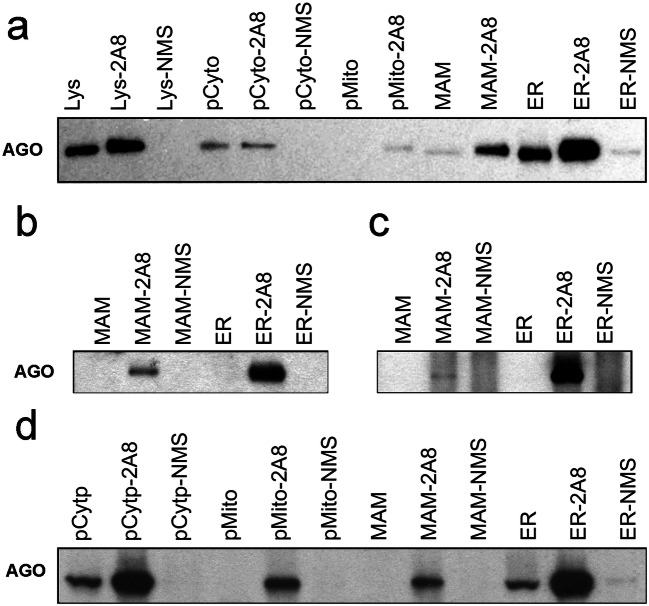
Immunoprecipitation and Western blot analysis detection of AGO protein. Human frontal cortex samples from 3 individual cases (**a** (case 3), **b** (case 1), and **c** (case 5)) and rat cortical tissue (**d**) were subjected to subcellular fractionation followed by immunoprecipitation (IP) with either anti-AGO (*2A8*, generated in mouse) or normal mouse serum (*NMS*). The immunoprecipitation products were analyzed by Western blotting using a rabbit anti-AGO antibody (Cell Signaling). AGO was found in all of the 2A8 immunoprecipitation subcellular fractionation samples but not in samples incubated with NMS. Incubation with NMS was not conducted on every mitochondria (*pMito*) and *MAM* samples due to the relatively low abundance of protein in these two subcellular fractions. *Lys*, post-nuclear total lysate; *Lys-2A8*, post-nuclear total lysate IP with anti-AGO 2A8 antibody; *Lys-NMS*, post-nuclear total lysate IP with NMS; *pCyto*, cytosol; *pCyto-2A8*, cytosol IP with anti-AGO 2A8 antibody; *pCyto-NMS*, cytosol IP with NMS; *pMito*, mitochondria lysate; *pMito-2A8*, mitochondria lysate IP with anti-AGO 2A8 antibody; *pMito-NMS*, mitochondria lysate IP with NMS; *MAM*, total MAM lysate; *MAM-2A8*, MAM lysate IP with anti-AGO 2A8 antibody; *MAM-NMS*, MAM lysate IP with NMS; *ER*, total ER lysate; *ER-2A8*, ER lysate IP with anti-AGO 2A8 antibody; *ER-NMS*, ER lysate IP with NMS

We then examined whether the miRNA executing complex AGO protein is also present in the MAMs. The subcellular fractions from three different human cases were subjected to immunoprecipitation (IP) with anti-AGO antibody or normal mouse IgG (NMS) as a control. The co-IPed proteins were then separated by SDS-PAGE, followed by the Western blot analysis using a different source of anti-AGO antibody (anti-AGO2, C34C6, Rabbit mAb, Cell Signaling Technology Cat no. 2897, RRID:AB_2096291; antibody dilution: 1:500). As shown in Fig. 6a, AGO protein was detected in IP-AGO of MAMs as well as mitochondria, cytosol, and ER but not in control NMS. The presence of AGO in the MAMs was confirmed in the two additional human cases (Fig. 6b and c), as well as by mass spectrometry analysis (Suppl. File 1).

Finally, IP experiments using rat brain subcellular fractions showed that AGO was also present in MAMs as well as cytosol, mitochondria, and ER (Fig. 6d).

### MiRNAs Are Enriched in AGO Complexes Co-immunoprecipitated in MAM Fractions

MAM fractions from human or rat cortices were subjected to co-IP with anti-AGO antibody or normal mouse IgG (NMS) as a control. Bound RNAs were isolated and subjected to single-tube RT-qPCR. The analysis revealed that miRNAs were enriched in AGO co-IP compared with NMS in both human and rat brain tissues (Table 2). Note that the level of miRNA enrichment in AGO co-IPs were different in each subcellular fraction and in separate human cases or rats. This is most likely the result of unequal protein inputs in each IP, but might also reflect biological differences between the individual persons and animals.

### Changes in Mitochondria/MAM-Enriched miRNAs Following Mitochondria Uncoupling or TBI

**Fig. 7:**
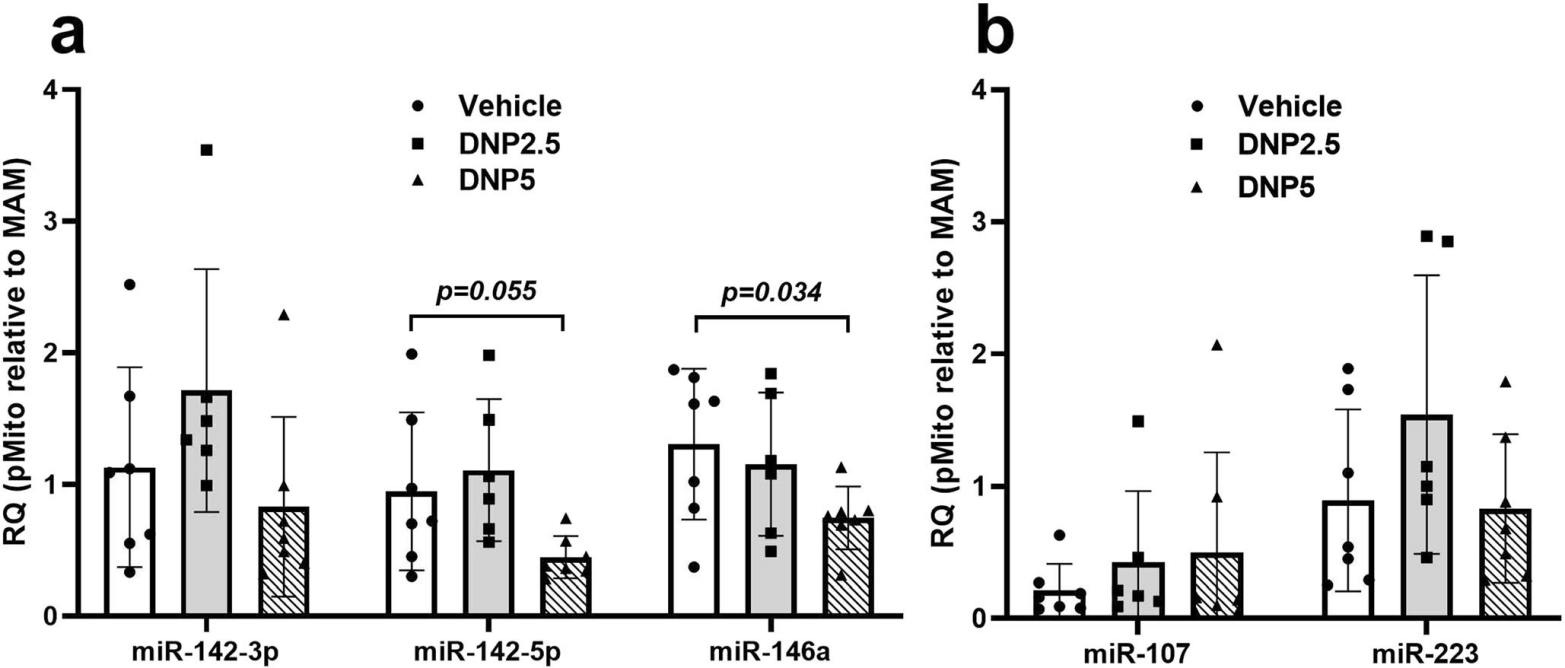
Effects of mild mitochondrial uncoupling on the enrichments of miRNAs in mitochondria and MAM fractions. The effects of different concentrations of uncoupling agent 2,4-dinitrophenol (DNP) on mitochondria-enriched miRNAs (**a**) and on the miRNAs that were not enriched in mitochondria (**b**) (*n* = 7 of rats in each group. One-way ANOVA and 2-sample unpaired Student’s *t* test). The relative quantity (RQ) of miRNAs in each group was expressed as RQ = 2^−(Ct-Mito − Ct-MAM)^ (RQ equals 2 to the power of negative (Ct-Mito minus Ct-MAM). *Vehicle*: DMSO; *DNP2.5*: 2.5 mg/kg DNP treatment; *DNP5*: 5 mg/kg DNP treatment

Mitochondria dysfunction is associated with a number of acute and chronic neurologic disease states including TBI [63, 64]. Therefore, we tested whether events that alter mitochondrial function impacts the inter-organelle distribution of mitochondria-associated miRNAs. In the first set of experiments, systemic treatment of rats with 2,4-DNP was used to induce mild mitochondrial uncoupling, which transiently disconnects the flow of electrons through the electron transport chain from the production of ATP [65]. At 3 h following 2,4-DNP treatment, we observed a concentration-dependent reduction of mitochondria-enriched miRNAs in the mitochondria (Fig. 7). Specifically, in the group treated with the higher concentration of 2,4-DNP (5 mg/kg), the levels of miR-146a decreased in mitochondria relative to MAM by 1.75-fold (*p* = 0.034, *d* = 0.97) (one-way ANOVA and 2-sample unpaired *t* test, df = 12). Although not statistically significant, the levels of miR-142-3p and miR-142-5p (2.1-fold, *p* = 0.055, *d* = 0.87) also decreased in the mitochondrial fraction (Fig. 7a). We did not observe any significant changes of miRNAs (e.g., miR-107 and miR-223) that were not enriched in rat brain mitochondria (Fig. 7b).

**Fig. 8:**
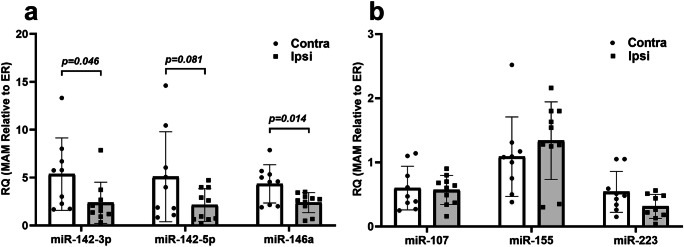
Effects of traumatic brain injury (TBI) on the enrichments of miRNAs in MAM and ER fractions. Twenty-four hours following a severe TBI (*n* = 10 rats), rat cortical tissues were subjected to subcellular fractionation and miRNAs were quantified by RT-qPCR. Effects of TBI on mitochondria-enriched miRNAs (**a**), and other miRNAs that are not enriched in the mitochondrial fraction (**b**) (2-sample unpaired Student’s *t* test). The relative quantity (RQ) of miRNAs in each group was expressed as RQ = 2^−(Ct-MAM − Ct-ER)^ (RQ equals 2 to the power of negative (Ct-MAM minus Ct-ER). *Contra*, contralateral cortices (uninjured); *Ipsi*, ipsilateral cortices (injured)

In a second set of experiments, we examined the subcellular miRNA distribution pattern in the cortex of rats at 24 h following a severe CCI injury. While no significant miRNA changes were observed between mitochondria and MAMs for the mitochondria/MAM-enriched miRNAs (miR-142-3p, miR-142-5p, and miR-146a), TBI resulted in a change of MAM to ER ratio in all three miRNAs (miR-146a, *p* = 0.014, *d* = 0.86), miR-142-3p (*p* = 0.046, *d* = 0.68), and miR-142-5p (*p* = 0.081, *d* = 0.59), in the ipsilateral cortex relative to uninjured contralateral cortex (2-sample unpaired *t* test, df = 18) (Fig. 8a). As observed in the uncoupling experiments above, there were no significant changes in miRNAs that are not enriched in the mitochondrial or MAM fraction, including miR-107, miR-155, and miR-223 (Fig. 8b).

## Discussion

We show here for the first time that MAMs are subcellular locations for miRNAs in rat and human cerebral cortex, and more specifically, this cellular domain contains several inflammatory-responsive miRNAs that are also highly enriched in the mitochondria. Our prior studies showed that, relative to the cytosol, miR-146a, miR-142-3p, and miR-142-5p are highly enriched in mitochondria isolated from rat hippocampus and postulated the presence of an inter-organelle network for miRNA localization and trafficking [29, 31]. We now report that these same three miRNAs, together with another inflammatory-responsive miRNA, miR-223, are also enriched in mitochondria isolated from human cerebral cortex gray matter, suggesting a conserved biological function. Moreover, we found that the levels of these inflammatory-responsive miRNAs were much higher in MAMs relative to ER and cytosol, and that mild uncoupling or cortical injury in rats results in the re-distribution of these miRNAs within what we hypothesize to be a mitochondria–MAM–ER compartmental network. Taken together, these results suggest that the MAMs may function as a node for inter-organelle miRNA shuttling between the mitochondria and ER and may serve as a subcellular locus for inflammatory gene regulation in stressful conditions.

Eukaryotic cells are highly compartmentalized with specialized functions subserved by membranous organelles. These organelles engage in dynamic contact and are in constant communication with each other. This “crosstalk” provides a network for enabling essential cellular functions in response to a broad range of stimuli. The mitochondria and ER contact sites designated as MAMs serve as a platform to accommodate the communication between both organelles and assist in efficient responses to changing cellular needs.

To our knowledge, there are no published studies demonstrating the distribution of miRNAs in subcellular fractions obtained from human brain. Our miRNA TLDA data revealed a specific differential distribution pattern of numerous miRNAs in the mitochondria, MAMs, ER, and cytosol isolated from human neocortex. For example, we found that miR-124a was preferentially expressed in cytosol, and that miR-146a, miR-142-3p, miR-142-5p, miR-21, miR-27a, and miR-223 were highly associated with mitochondria and MAMs. These mitochondria and MAM-enriched miRNAs have been reported to be involved in regulating inflammatory and immune responses, and/or autophagy [66, 67], which is particularly intriguing, given the role of mitochondria and MAMs in these same cellular response events [4, 5, 9]. The distinct miRNA localization patterns suggest that specific organelles may play diverse roles in individual miRNA activities such as targeting, translocation and trafficking, and/or metabolic turnover. The cell type specificity of the miRNA responses we are studying are not completely known but seem to pertain to cells with strong roles in neuroinflammation. More specifically, our previous study shows that miR-146a, miR-142-3p, miR-142-5p, and miR-223 are predominately expressed in microglia and astrocytes [31], which is in line with the functional role of these miRNAs. However, considering the morphological heterogeneity and dynamic nature of mitochondria in different tissues and cell types, the mitochondria and ER interactions are complex [68]. Thus, for example, the distribution and the MAM-associated miRNAs may vary among cell types. Another interesting question is whether MAM-enriched miRNAs directly participate in MAM-mediated biological process, such as phospholipid biosynthesis, cholesterol esterification, and calcium transport [1, 10, 12, 21]. Notably, MAM-enriched miR-223 targets methylsterol monooxygenase 1 (MSMO1), 3-hydroxy-3-methyl-glutaryl-coenzyme A reductase (HMGCR), and a scavenger receptor, class B type 1 (SR-B1), which are involved in cholesterol biosynthesis and lipoprotein synthesis [69]. It remains to be seen how the enrichment of miR-223 in MAM affects these MAM-mediated biological process, and future studies are required to understand the cell/tissue-specificity of MAM-associated miRNAs and their functional roles in normal and pathological conditions.

The presence of miRNAs and their functional executing protein AGO in the MAM fraction suggests that the MAM-associated miRNAs are, in some way(s), operational. The AGO antibody 2A8 that we used in this study was raised in mice immunized with recombinant human AGO2 protein, but the 2A8 antibody recognizes all 4 human AGO proteins (AGO1, AGO2, AGO3, and AGO4) [47]. In our study, AGO1 was the AGO species identified by the mass spectrometry from anti-AGO 2A8 co-immunoprecipitation of the MAM fraction (Suppl. File 1). Both AGO1 and AGO2 are involved in transcriptional gene regulation [70] with only AGO2 possessing mRNA cleavage activity. It will be necessary to further investigate the functional role of MAM/mitochondria-associated AGO complexes in gene regulation and how the miRNA-AGO complexes traffic between the organelles in response to cellular demands.

The altered enrichment of miRNAs in specific organelles may reflect a microdomain-specific signaling mechanism allowing for rapid cellular responses to events impacting mitochondrial function. Mitochondria uncoupling is the process by which protons in the mitochondrial inner membrane space are translocated into the matrix, effectively reducing the mitochondrial membrane potential. In our study, we found that mitochondria uncoupling using 2,4-DNP led to a significant shift in the levels of miR-146a from the mitochondria to the MAMs. There also was a trend for a shift of miR-142-5p and miR-142-3p from the mitochondria to the MAMs, but this did not reach statistical significance. Following experimental TBI, there is a rapid and significant loss of mitochondrial bioenergetics, among other detrimental cellular events, that contributes to cellular dysfunction [41, 64, 71]. We found that 24 h after a severe TBI, there was a substantial inter-organelle shift in the same inflammatory-responsive miRNAs. However, in this case where mitochondrial function was severely compromised (relative to uncoupling), the shift in these miRNAs was from the MAMs to the ER. Interestingly, we did not find any evidence supporting the inter-organelle shift of other miRNAs such as miR-107, or miR-155, although these cytosolic miRNAs have been shown to be altered in TBI [31, 72]. There are at least two possibilities for why we did not observe a significant shift of mitochondria/MAM-enriched miRNAs from mitochondria to MAM. First, using the contralateral uninjured brains as controls may mask the true effects of CCI since the contralateral side of the brain may respond to signals transduced from the injured ipsilateral side of the brain. Second, the single time point (24 h post-CCI) we examined may not capture the translocation of miRNAs that might occur at earlier or later time points. Nevertheless, based on current findings, we hypothesize that mitochondria play a role in driving the translocation of these select miRNAs to other subcellular compartments in response to cellular demands and stressors. It is possible that the observed alteration of miRNA organelle enrichment following uncoupling and CCI was independent of mitochondria function. For example, it could be a result of direct modification of the mitochondria–ER contacts. Future studies may determine whether the mitochondria/MAM-enriched miRNAs are translocated from mitochondria to MAMs and then to the ER, or if they are recruited from some other cellular compartment.

It should be noted that these experiments were performed in male rats at one single time point, which may not take into account the actual sequence of biological events and the impact of sex differences, especially under stress or pathological conditions. However, our goal in this initial study was to reveal the intra-organelle trafficking of miRNAs under various conditions that impact mitochondrial function. Further understanding on how miRNAs translocate within the organelles will benefit from a more comprehensive design of the studies including different time points, additional controls, and examining both male and female animals.

The localization and re-distribution of certain inflammatory miRNAs in organelles suggest the presence of a novel and dynamic miRNA-based mechanism for regulating cellular inflammatory responses. For example, miR-146a is a key miRNA involved in the innate immune and inflammatory responses, and helps regulate the production of inflammatory cytokines [73]. Initially identified as an NF-κB-induced miRNA, miR-146a helps modify cytokine signaling via a negative feedback mechanism, in part, by targeting *Traf6* and *Irak1*. Previous studies have shown that miR-146a deficiency in mice caused autoimmunity and a hyper-reactive immune response [74]. Intriguingly, miR-146a-knockout mice also showed constitutive activation of the NF-κB pathway, with excessive inflammation and tumorigenesis during aging [75]. Our findings that miR-146a is preferentially localized in mitochondria and MAMs and responds to cellular demands by altering the enrichment suggests a novel mechanism for specific gene regulation in the NF-κB pathway perhaps by targeting *Traf6* and *Irak1*.

While impairment or perturbation of MAM function has been implicated in several neurodegenerative disease conditions [13–20, 76], the contributing underlying mechanism(s) remain unknown. Relevant to our study, miR-146a has been specifically implicated in AD [77–80] and other neurodegenerative diseases [81, 82]. Our observations that specific inflammatory-responsive miRNAs are preferentially localized in the MAMs, and re-distributed under particular experimental conditions, points to yet another level of complexity related to post-transcriptional gene regulation in the brain. There are several possible scenarios: (1) miRNAs located in MAMs may target specific transcripts that co-localize in the MAMs. Our immunoprecipitation and mass spectrometry results revealed that AGO1 protein is present in MAM fractions and enriched with miRNAs, implying the existence of a functional miRNA ribonucleoprotein complex. (2) MAMs may serve as a miRNA trafficking hub between mitochondria and ER, as well as other cytoplasmic granules and organelles. Our experiments show that events affecting mitochondria function (e.g. mitochondria uncoupling and TBI), alter the distribution of specific miRNAs from one organelle to another. (3) MAMs may serve as a platform for incorporating novel signaling molecules into miRNA/AGO complexes for specific targeting. For example, the MAMs have been shown to play key roles in autophagosome and inflammasome assembly [4, 5, 11]. Interestingly, assembly of the NLRP3 inflammasome involves physical interactions with the mitochondria [5] as well as NF-κB activation [83], which is regulated, in part, by miR-146a activity [73]. While MAMs presumably have currently unknown roles, our study provides strong and highly novel evidence for the importance of MAM function in the activity of a subset of miRNAs. At the present time, understanding the role(s) of inflammatory-responsive miRNAs located in the mitochondria and MAMs is very limited. As such, future studies investigating miRNA–mitochondria–MAM–ER interactions will be necessary to link inter-organelle miRNA trafficking to physiological and pathological processes, and to gain better understanding about how modifying this “crosstalk” might contribute to more effective strategies in the treatment of brain diseases.

## Electronic Supplementary Material

Supplementary file 1 (PDF 60 kb)

Supplementary file 2 (PDF 76 kb)

Supplementary table 1 (PDF 44 kb)

Supplementary table 2 (PDF 43 kb)

## Abbreviations

AD: Alzheimer’s disease
AGO: Argonaute protein
ALS: Amyotrophic lateral sclerosis
CCI: Controlled cortical impact
DMSO: Dimethyl sulfoxide
DTT: Dithiothreitol
ER: Endoplasmic reticulum
*Gapdh*: Glyceraldehyde 3-phosphate dehydrogenase
Grp75: Glucose-regulated protein 75
IAA: Iodoacetamide
IP: Immunoprecipitation
MAM: Mitochondria-associated ER membrane
miRNA: MicroRNA
MIQE: Minimum Information for Publication of Quantitative Real-Time PCR Experiments
NDUFA9: NADH dehydrogenase [ubiquinone] 1 alpha subcomplex subunit 9
NMS: Normal mouse IgG
PD: Parkinson’s disease
PDZD8: PDZ domain-containing protein 8
PMI: Postmortem intervals
RRID: Research Resource Identifier
TBI: Traumatic brain injury
DNP: 2,4-Dinitrophenol
TLDA: TaqMan® Low-Density Array
UK-ADC: University of Kentucky Alzheimer’s Disease Center
