# Supplementary material for: The Mitochondria-Associated ER Membranes Are Novel Subcellular Locations Enriched for Inflammatory-Responsive MicroRNAs
Source: Mol Neurobiol. 2020 May 25;57(7):2996–3013. doi: 10.1007/s12035-020-01937-y (PMC7320068; doi:10.1007/s12035-020-01937-y)
Supplement: Supplementary file 1 — (PDF 60 kb) [file 12035_2020_1937_MOESM1_ESM.pdf]

Suppl File 1

Accession	Q9UL18
Description	Protein argonaute-1 OS=Homo sapiens GN=AGO1 PE=1 SV=3 - [AGO1_HUMAN]
Score	32.73
Coverage	1.40
# Proteins	1
# Unique Peptides	1
# Peptides	1
# PSMs	1
# AAs	857
MW [kDa]	97.2
calc. pI	9.16
Location	
GO	

Confidence Level	High
Sequence	RPGIGTVGKPIK
PSM Ambiguity	Unambiguous
# Proteins	1
# Protein Groups	1
Protein Group Accessions	Q9UL18
Modifications	
Activation Type	CID
ΔScore	1.0000
ΔCn	0.0000
Rank	1
Search Engine Rank	1
IonScore	33
Exp Value	0.000532072
# Missed Cleavages	0
Isolation Interference [%]	30
Ion Inject Time [ms]	100
Charge	3
m/z [Da]	408.25934
MH+ [Da]	1222.76346
ΔM [ppm]	0.30
RT [min]	16.65
First Scan	2360
Last Scan	2360
MS Order	MS2
Ions Matched	9/110
Spectrum File	05312018_Wang_WX_A1.raw
