# Supplementary material for: The Mitochondria-Associated ER Membranes Are Novel Subcellular Locations Enriched for Inflammatory-Responsive MicroRNAs
Source: Mol Neurobiol. 2020 May 25;57(7):2996–3013. doi: 10.1007/s12035-020-01937-y (PMC7320068; doi:10.1007/s12035-020-01937-y)
Supplement: Supplementary file 2 — (PDF 76 kb) [file 12035_2020_1937_MOESM2_ESM.pdf]

Suppl File 2

Assay Name	Assay ID	Assay Target Sequence	miRBase ID (v20)
hsa-let-7a	000377	UGAGGUAGUAGGUUGUAUAGUU	hsa-let-7a-5p
hsa-let-7b	002619	UGAGGUAGUAGGUUGUGUGGUU	hsa-let-7b-5p
hsa-let-7c	000379	UGAGGUAGUAGGUUGUAUGGUU	hsa-let-7c-5p
hsa-let-7f	000382	UGAGGUAGUAGAUUGUAUAGUU	hsa-let-7f-5p
hsa-miR-103	000439	AGCAGCAUUGUACAGGGCUAUGA	hsa-miR-103a-3p
hsa-miR-106a	002169	AAAAGUGCUUACAGUGCAGGUAG	hsa-miR-106a-5p
hsa-miR-107	000443	AGCAGCAUUGUACAGGGCUAUCA	hsa-miR-107
hsa-miR-125b	000449	UCCCUGAGACCCUAACUUGUGA	hsa-miR-125b-5p
hsa-miR-1274B	002884	UCCCUGUUCGGGCGCCA	NA
hsa-miR-1298	002861	UUCAUUCGGCUGUCCAGAUGUA	hsa-miR-1298-5p
hsa-miR-132	000457	UAACAGUCUACAGCCAUGGUCG	hsa-miR-132-3p
hsa-miR-142-3p	000464	UGUAGUGUUUCCUACUUUAUGGA	hsa-miR-142-3p
hsa-miR-142-5p	002248	CAUAAAGUAGAAAGCACUACU	hsa-miR-142-5p
hsa-miR-146a	000468	UGAGAACUGAAUUCCAUGGGUU	hsa-miR-146a-5p
hsa-miR-146b	001097	UGAGAACUGAAUUCCAUAGGCU	hsa-miR-146b-5p
hsa-miR-150	000473	UCUCCCAACCCUUGUACCAGUG	hsa-miR-150-5p
hsa-miR-155	002623	UUAAUGCUAAUCGUGAUAGGGGU	hsa-miR-155-5p
hsa-miR-15a	000389	UAGCAGCACAUAAUGGUUUGUG	hsa-miR-15a-5p
hsa-miR-15b	000390	UAGCAGCACAUCAUGGUUUACA	hsa-miR-15b-5p
hsa-miR-16	000391	UAGCAGCACGUAAAUAUUGGCG	hsa-miR-16-5p
hsa-miR-17	002308	CAAAGUGCUUACAGUGCAGGUAG	hsa-miR-17-5p
hsa-miR-181a	000480	AACAUUCAACGCUGUCGGUGAGU	hsa-miR-181a-5p
hsa-miR-181c	000482	AACAUUCAACCUGUCGGUGAGU	hsa-miR-181c-5p
hsa-miR-195	000494	UAGCAGCACAGAAAUAUUGGC	hsa-miR-195-5p
hsa-miR-19b	000396	UGUGCAAAUCCAUGCAAAACUGA	hsa-miR-19b-3p
hsa-miR-204	000508	UUCCCUUUGUCAUCCUAUGCCU	hsa-miR-204-5p
hsa-miR-20a	000580	UAAAGUGCUUAUAGUGCAGGUAG	hsa-miR-20a-5p
hsa-miR-21	000397	UAGCUUAUCAGACUGAUGUUGA	hsa-miR-21-5p
hsa-miR-223	002295	UGUCAGUUUGUCAAAUACCCCA	hsa-miR-223-3p
hsa-miR-24	000402	UGGCUCAGUUCAGCAGGAACAG	hsa-miR-24-3p
hsa-miR-27a	000408	UUCACAGUGGCUAAGUUCCGC	hsa-miR-27a-3p
hsa-miR-29a	002112	UAGCACCAUCUGAAAUCGGUUA	hsa-miR-29a-3p
hsa-miR-29b	000413	UAGCACCAUUUGAAAUCAGUGUU	hsa-miR-29b-3p
hsa-miR-29c	000587	UAGCACCAUUUGAAAUCGGUUA	hsa-miR-29c-3p
hsa-miR-30a-3p	000416	CUUUCAGUCGGAUGUUUGCAGC	hsa-miR-30a-3p
hsa-miR-30a-5p	000417	UGUAAACAUCCUCGACUGGAAG	hsa-miR-30a-5p
hsa-miR-30b	000602	UGUAAACAUCCUACACUCAGCU	hsa-miR-30b-5p
hsa-miR-30c	000419	UGUAAACAUCCUACACUCUCAGC	hsa-miR-30c-5p
hsa-miR-30d	000420	UGUAAACAUCCCCGACUGGAAG	hsa-miR-30d-5p
hsa-miR-30e-3p	000422	CUUUCAGUCGGAUGUUUACAGC	hsa-miR-30e-3p
hsa-miR-34a	000426	UGGCAGUGUCUUAGCUGGUUGU	hsa-miR-34a-5p
hsa-miR-34b	002102	CAAUCACUAACUCCACUGCCAU	hsa-miR-34b-3p
hsa-miR-34c	000428	AGGCAGUGUAGUUAGCUGAUUGC	hsa-miR-34c-5p
hsa-miR-497	001043	CAGCAGCACACUGUGGUUUGU	hsa-miR-497-5p
hsa-miR-9	000583	UCUUUGGUUAUCUAGCUGUAUGA	hsa-miR-9-5p
mmu-miR-124a	001182	UAAGGCACGCGGUGAAUGCC	hsa-miR-124-3p
mmu-miR-451	001141	AAACCGUUACCAUUACUGAGUU	hsa-miR-451a
U6 snRNA	001973	GTGCTCGCTTCGGCAGCACATATACTAAAA	U6 snRNA
Gapdh	Rn01775763		
