# Supplementary material for: The Mitochondria-Associated ER Membranes Are Novel Subcellular Locations Enriched for Inflammatory-Responsive MicroRNAs
Source: Mol Neurobiol. 2020 May 25;57(7):2996–3013. doi: 10.1007/s12035-020-01937-y (PMC7320068; doi:10.1007/s12035-020-01937-y)
Supplement: Supplementary file 3 — (PDF 44 kb) [file 12035_2020_1937_MOESM3_ESM.pdf]

Suppl. Table 1. Single-tube TaqMan® miRNA analysis of subcellular fractions isolated from autopsied human frontal cortices

	miR-107					miR-124a					miR-142-3p					miR-142-5p					miR-146a					miR-223				
**Case**	**C1**	**C2**	**C3**	**C4**	**C5**	**C1**	**C2**	**C3**	**C4**	**C5**	**C1**	**C2**	**C3**	**C4**	**C5**	**C1**	**C2**	**C3**	**C4**	**C5**	**C1**	**C2**	**C3**	**C4**	**C5**	**C1**	**C2**	**C3**	**C4**	**C5**
**pCyto**	1.0	1.0	1.0	1.0	1.0	1.0	1.0	1.0	1.0	1.0	1.0	1.0	1.0	1.0	1.0	1.0	1.0	1.0	1.0	1.0	1.0	1.0	1.0	1.0	1.0	1.0	1.0	1.0	1.0	1.0
**pMito**	0.9	1.0	0.6	0.1	0.4	0.7	0.9	1.4	0.1	0.1	14.4	10.4	8.5	2.1	5.5	5.1	6.7	6.8	2.8	4.6	4.8	3.1	5.5	1.8	4.5	19.8	5.7	23.1	4.2	7.7
**MAM**	4.1	0.6	7.6	1.2	3.4	2.6	0.6	0.2	1.2	1.9	9.0	0.1	10.9	5.5	9.0	12.8	0.2	8.3	10.1	6.8	10.4	0.6	8.0	6.8	8.1	35.4	0.3	30.1	12.9	8.6
**ER**	3.7	0.0	2.1	1.3	2.5	3.1	0.0	0.1	1.4	1.3	4.3	0.3	2.0	2.8	3.9	4.0	0.7	1.5	2.2	1.9	3.3	0.9	1.3	4.1	3.3	5.6	1.0	4.2	3.6	3.8
