# Supplementary material for: The Mitochondria-Associated ER Membranes Are Novel Subcellular Locations Enriched for Inflammatory-Responsive MicroRNAs
Source: Mol Neurobiol. 2020 May 25;57(7):2996–3013. doi: 10.1007/s12035-020-01937-y (PMC7320068; doi:10.1007/s12035-020-01937-y)
Supplement: Supplementary file 4 — (PDF 43 kb) [file 12035_2020_1937_MOESM4_ESM.pdf]

Suppl. Table 2. Single-tube TaqMan® miRNA analysis of subcellular fractions isolated from rat cortices.

	miR-107					miR-142-3p					miR-142-5p					miR-146a					miR-223				
**Rats**	**R1**	**R2**	**R3**	**R4**	**R5**	**R1**	**R2**	**R3**	**R4**	**R5**	**R1**	**R2**	**R3**	**R4**	**R5**	**R1**	**R2**	**R3**	**R4**	**R5**	**R1**	**R2**	**R3**	**R4**	**R5**
**pCyto**	1.0	1.0	1.0	1.0	1.0	1.0	1.0	1.0	1.0	1.0	1.0	1.0	1.0	1.0	1.0	1.0	1.0	1.0	1.0	1.0	1.0	1.0	1.0	1.0	1.0
**pMito**	0.1	0.1	0.5	0.1	0.0	2.3	7.6	3.5	4.4	2.1	5.3	9.9	3.7	7.5	3.0	18.7	14.2	18.5	14.5	3.2	0.5	1.4	0.9	0.5	0.2
**MAM**	0.4	0.7	3.3	1.2	1.1	2.1	3.0	3.1	7.1	6.1	3.6	5.0	5.2	16.7	19.7	10.0	8.8	10.2	17.7	12.9	0.3	0.7	3.1	1.0	1.5
**ER**	3.6	0.8	11.6	2.1	0.7	2.9	0.7	6.3	2.3	1.9	1.6	0.3	3.4	1.2	2.4	3.0	0.9	6.6	1.8	1.4	1.9	0.7	4.9	1.6	0.5
